# Structure-Based Discovery of Small Molecule Inhibitors of Cariogenic Virulence

**DOI:** 10.1038/s41598-017-06168-1

**Published:** 2017-07-20

**Authors:** Qiong Zhang, Bhavitavya Nijampatnam, Zhang Hua, Thao Nguyen, Jing Zou, Xia Cai, Suzanne M. Michalek, Sadanandan E. Velu, Hui Wu

**Affiliations:** 1 Department of Pediatric Dentistry, University of Alabama at Birmingham, School of Dentistry, Birmingham, Alabama 35294 USA; 2 Department of Pediatric Dentistry, West China Hospital of Stomatology, State Key Laboratory of Oral Diseases, Sichuan University, Chengdu, Sichuan 610041 P.R. China; 3 Department of Chemistry, University of Alabama at Birmingham, 901, 14th Street S, Birmingham, AL 35294 USA; 4 Department of Microbiology, University of Alabama at Birmingham, Birmingham, AL 35294 USA

## Abstract

*Streptococcus mutans* employs a key virulence factor, three glucosyltransferase (GtfBCD) enzymes to establish cariogenic biofilms. Therefore, the inhibition of GtfBCD would provide anti-virulence therapeutics. Here a small molecule library of 500,000 small molecule compounds was screened *in silico* against the available crystal structure of the GtfC catalytic domain. Based on the predicted binding affinities and drug-like properties, small molecules were selected and evaluated for their ability to reduce *S. mutans* biofilms, as well as inhibit the activity of Gtfs. The most potent inhibitor was further characterized for Gtf binding using OctetRed instrument, which yielded low micromolar K_D_ against GtfB and nanomolar K_D_ against GtfC, demonstrating selectivity towards GtfC. Additionally, the lead compound did not affect the overall growth of *S. mutans* and commensal oral bacteria, and selectively inhibit the biofilm formation by *S. mutans*, indicative of its selectivity and non-bactericidal nature. The lead compound also effectively reduced cariogenicity i*n vivo* in a rat model of dental caries. An analog that docked poorly in the GtfC catalytic domain failed to inhibit the activity of Gtfs and *S. mutans* biofilms, signifying the specificity of the lead compound. This report illustrates the validity and potential of structure-based design of anti-*S. mutans* virulence inhibitors.

## Introduction

Dental caries is a multifactorial disease of bacterial origin, which is characterized by the localized destruction of dental hard tissues^1, 2^. Though the oral cavity harbors over 700 different bacterial species, *Streptococcus mutans* initiates the cariogenic process and remains as the key etiological agent^3^. Using key matrix producing enzymes, glucosyltransferases (Gtfs), *S. mutans* produces sticky glucosyl glucan polymers, which facilitate the attachment of the bacteria to the tooth surface. The glucans is a major component of the biofilm matrix that shields the microbial community from host defenses, mechanical and oxidative stresses, and orchestrates the formation of cariogenic biofilms^4^. Furthermore, copious amounts of lactic acid are produced as a byproduct of bacterial consumption of dietary sugars within the mature biofilm community, which ultimately leads to demineralization of the tooth surface, ensuing cariogenesis.

Current practices to prevent dental caries remove oral bacteria non-discriminatively through chemical and physical means such as mouthwash and tooth brushing^5^. Since the biofilm assembly renders bacteria to become more resistant to antibiotics and other manipulations, these traditional approaches have had only limited success. Additionally, existing mouthwashes are often associated with adverse side effects because the use of broad-spectrum antimicrobials are often detrimental to beneficial commensal species.

Selectively targeting cariogenic pathogens such as *S. mutans* has been explored previously, however it was found that the antimicrobial peptide also alters the overall microbiota^6^. Our increasing understanding of bacterial virulence mechanisms provides new opportunities to target and interfere with crucial virulence factors such as Gtfs. This approach has the added advantages of not only being selective, but may also help to preserve the natural microbial flora of the mouth^7^, which may avoid to exert the strong pressure to promote the development of antibiotic resistance, overcoming a major public health issue in the antibiotic era. It is well established that glucans produced by *S. mutans* Gtfs contribute significantly to the cariogenicity of dental biofilms. Therefore, the inhibition of the Gtf activity and the consequential glucan synthesis would impair the *S. mutans* virulence, which could offer an alternative strategy to prevent and treat biofilm-related diseases^8, 9^.

*S*. *mutans* harbors three Gtfs: GtfB, GtfC, and GtfD. While GtfB synthesizes pre-dominantly insoluble glucans, GtfD only produces water-soluble glucans, and GtfC can synthesize both soluble and insoluble glucans^10–12^. Previous studies have demonstrated that glucans produced by GtfB and GtfC are essential for the assembly of the *S. mutans* biofilms^4^, while glucans produced by GtfD serve not only as a primer for GtfB, but also as a source of nutrient for *S. mutans* and other bacteria^13, 14^. All Gtfs are composed of three functional regions: the N-terminal variable junction region, the C-terminal glucan-binding region, and the highly conserved catalytic region in the middle, which is essential for the glucan synthesis. The crystal structural of GtfC from *S. mutans* has been determined^15^, which provides key molecular insights for the design and development of novel Gtf inhibitors.

Polyphenolic compounds^16–23^ that include catechins, flavonoids, proanthocyanidin oligomers, and other plant-derived analogs^24, 25^ and synthetic small molecules^26^ have been studied extensively for years and were found to display modest anti-biofilm activities through modulating the expression of Gtfs of *S. mutans*. However, the selectivity of these bioactive compounds remains to be determined and the potency is not satisfactory for the biofilm inhibition.

In the present study, novel inhibitors of *S. mutans* Gtfs were developed through *in silico* screening of commercial compound libraries against the active site of the catalytic domain from the *S. mutans* GtfC. A lead compound targeting Gtfs was identified, synthesized, and shown to have the ability to bind to Gtfs and inhibit *S. mutans* biofilm formation selectively *in vitro*. Furthermore, the lead compound possesses anti-virulence properties *in vivo*.

## Results

### Structure-based virtual screening to identify small-molecule compounds that target Gtfs and inhibit biofilm formation

**Figure 1:**
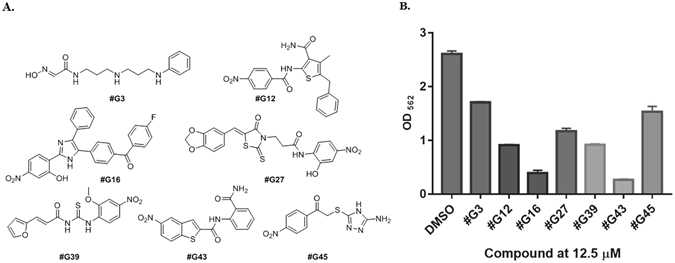
(**A**) Structures of seven most potent Gtf inhibitors of *S. mutans* biofilms. (**B**) Biofilm inhibitory activities of the potent inhibitors at 12.5 µM as determined by the crystal violet assay.

Taking advantage of the available crystal structure of the GtfC catalytic domain complexed with acarbose, we conducted a structure-based *in silico* screening of 500,000 drug-like compounds using the FlexX/LeadIT software. The top ranked small molecules, as calculated using the binding energy scores in the FlexX software, were considered based on their binding pose, potential interactions with key residues, and ease of synthesis. Due to the abundance of polar residues in the GtfC active site, several of the top scored docking scaffolds contain aromatic rings, nitro groups, and polar functional groups such as amides and heteroatoms such as sulfur, etc. A total of 90 compounds with diverse scaffolds which vary in their functional groups, hydrophobicity, and H-bond accepting/donating capacity were then purchased and subjected to *in vitro* biofilm assays using cariogenic *S. mutans*. Seven potent low micromolar inhibitors were identified (Fig. 1A). Two of these compounds (#G16 and #G43) were the most potent, as they inhibited more than 85% of *S. mutans* biofilms at 12.5 μM (Fig. 1B). Compounds #G16 and #G43 share several functional groups including a nitro group, heterocyclic rings, and polar carbonyl functional property.

### Inhibition of Gtfs by lead compounds

**Figure 2:**
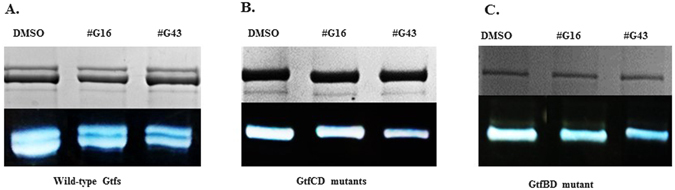
Gtf patterns of *S. mutans* UA159 and its mutant variants. Culture supernatants were prepared from *S. mutans* UA159 wild type and *gtf* double mutants, and then subjected to SDS-PAGE analysis with equivalent amount of proteins in each lane. The upper panel was stained with Coomassie blue to monitor the total protein amounts while the lower panel shows enzymatic activities of Gtfs with the treatment of the lead by the zymographic assay. The intensity of the bands were quantified using ImageJ in comparison to DMSO. (**A**) Effects of lead compounds #G16 and #G43 at 25 µM on the activity of Gtfs from wild type *S. mutans*. (**B**) Effects of lead compounds #G16 and #G43 at 25 µM on the activity of GtfB from *S. mutans* GtfCD mutants. (**C**) Effects of lead compounds #G16 and #G43 at 25 µM on the activity of GtfC from *S. mutans* GtfBD mutants.

Zymographic enzymatic assay was used to determine whether the lead compounds inhibited the activity of Gtfs that are responsible for the production of glucans and biofilm formation. Supernatants containing Gtf proteins prepared from *S. mutans* bacterial cultures were subjected to SDS-PAGE analysis and zymographic assay. Treatment of the SDS-PAGE gels with lead compounds #G16 and #G43 in a zymographic assay revealed that both #G16 and #G43 drastically reduced the glucan production by the Gtfs, #G43 was more potent (Fig. 2A, bottom panels). The same amount of the protein sample was used as controls and visualized by protein staining (Fig. 2A, top panels). The lead compounds were also tested against individual Gtfs using supernatant proteins harvested from cultures of various double mutants. Compound #G43 consistently inhibited the activity of both GtfB and GtfC (Fig. 2B and C), ImageJ analysis of the intensities suggest 80% inhibition of both enzymes, while compound #G16 had a smaller effect on the activity of GtfB (60% inhibition) (Fig. 2B) and GtfC (70% inhibition) (Fig. 2C). Overall #G43 is more potent than #G16 in inhibiting Gtfs.

### Binding kinetics of #G43 lead compound determined by OctetRed Analysis

**Figure 3:**
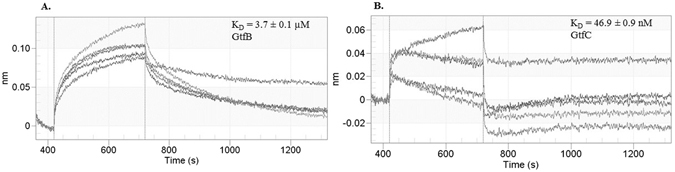
Binding curves of compound #G43 at varying concentrations with (**A**) GtfB, and (**B**) GtfC catalytic domain.

Zymograhic assays suggest that the lead compound #43 inhibited the activity of both GtfB and GtfC. To determine if the inhibition is attributed by the binding of the lead compound to the enzymes, the OctetRed96 system was used to characterize protein-small molecule binding kinetics. The his-tagged catalytic domains of GtfB and GtC were immobilized separately onto an anti-penta-HIS (HIS1K) biosensor which consists of high affinity, high specificity penta-his antibody pre-immobilized on a fiber optic biosensor. This sensor was then exposed to varying concentrations of #G43. Assay data fit to a 1:1 binding model with a fixed maximum response, which produced a K_D_ value of 3.7 µM for GtfB. The K_D_ value for GtfC was more potent at 46.9 nM (Fig. 3A and B). These data suggest that the lead compound is selective toward GtfC, the protein used in the *in-silico* analysis. It should be noted that the catalytic domain of GtfC is less soluble compared to that of GtfB’s, which may be responsible for the inherent higher off rate of the his-tag from the sensor, leading to a weaker association curve when compared to GtfB. Nevertheless, consistent nanomolar K_D_ values were obtained from three independent experiments.

### Expression of *gtf*s was not significantly affected by compound #G43

**Figure 4:**
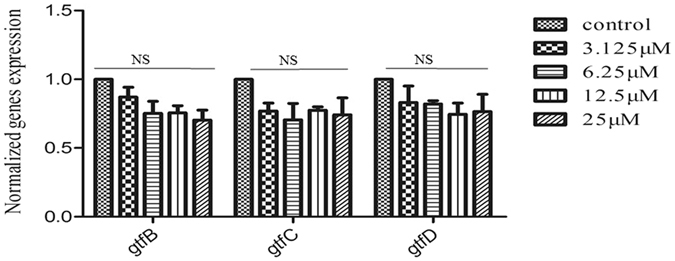
Effect of the compound #G43 on expression of *gtfs* in *S. mutans. S. mutans* UA159 cells treated with different concentrations of #G43 were harvested and used to prepare RNA. The expression of *gtfs* was examined by real time RT-PCR. The mRNA expression levels were calibrated by 16S rRNA. Values represent the means ± standard deviations from three independent experiments. NS indicates no significant difference between DMSO control and compound-treated groups. The P value > 0.05 is considered to be not significant.

We have also examined the effect of this potent small molecule on the gene expression of *gtf*s. The relative expression level of *gtf*s was evaluated by real time RT-PCR. Compared to the DMSO control group, expression of *gtf*s were marginally down-regulated after the treatment with compound #G43 at different concentrations. However, no significant difference was observed between the treated and control groups, suggesting that compound #G43 inhibited Gtfs via binding to the targets rather than altered expression of its targeting genes, *gtf*s (Fig. 4).

### The most potent compound is not bactericidal and did not inhibit the growth of commensal streptococcal species, and other oral bacteria

**Figure 5:**
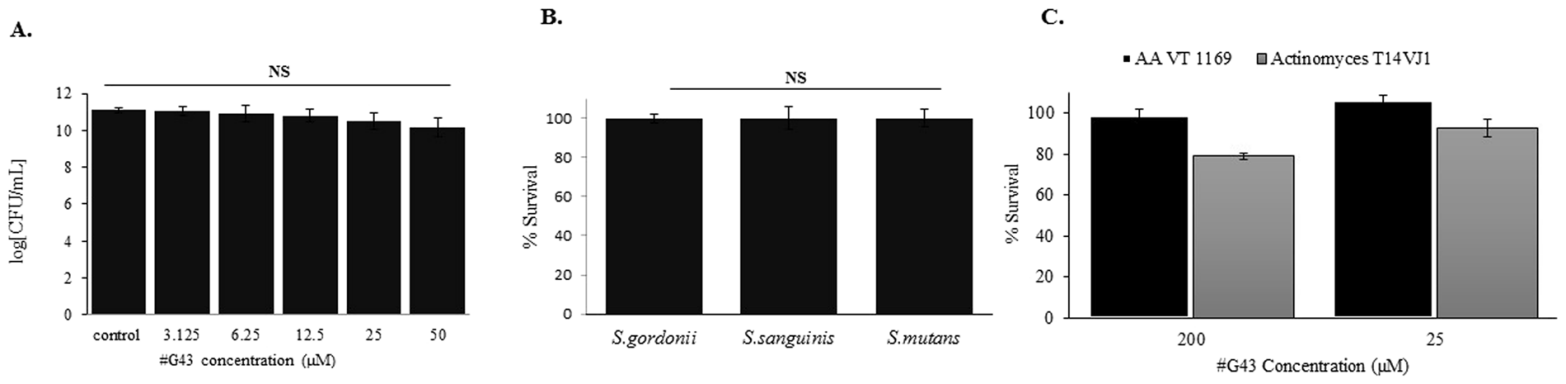
Effects of the lead compound #G43 on cell viability. (**A**) Effects on *S. mutans*. *S. mutans* was treated with DMSO and a serial dilution of #G43. The cell viability was determined by the numbers of CFU in a logarithmic scale. (**B**) Effects on commensal species. *S. gordonii*, *S. sanguinis*, and *S. mutans* were treated with 200 µM of the compound or DMSO, and bacterial growth was measured at OD_470_, and normalized to the DMSO control (100%). (**C**) Effects on *Aggregatibacter actinomycetemcomitans* and *Actinomyces naeslundii*. *A. actinomycetemcomitans* and *A. naeslundii* were treated with the lead compound at 200 µM or 25 µM or DMSO control, bacterial growth was measured at OD_470_, and normalized to the DMSO control (100%). Values represent the means ± standard deviations from three independent experiments. NS indicates that the cell viability between DMSO control and compound-treated groups was not significantly different. The P value > 0.05 is considered to be not significant.

To determine the selectivity of the lead compound toward *S. mutans* biofilm formation versus bacterial growth, we evaluated effects of the compound on bacterial growth and viability. No significant difference in *S. mutans* cell viability was observed between the control group and #G43 treated groups up to 200 µM (Fig. 5A), suggesting that the compound is not bactericidal towards *S. mutans*. This compound was also evaluated for its ability to inhibit two oral commensal streptococci: *S. sanguinis* and *S. gordonii* as the goal was to develop non-bactericidal and species-selective agents. The compound did not have any effect on bacterial growth (Fig. 5B) of both streptococcal species. In addition, we evaluated effects of the compound on other oral bacteria including *Aggregatibacter actinomycetemcomitans* VT1169, a Gram-negative, facultative anaerobe, and *Actinomyces naeslundii* T14VJ1, a gram-positive, facultative anaerobe (Fig. 5C). At 200 µM, the compound had no significant inhibition of *Aggregatibacter actinomycetemcomitans*. Slight inhibition (>20%) was observed of *Actinomyces naeslundii* growth at 200 µM, suggesting the selectivity towards *S. mutans*.

### #G43 did not inhibit the biofilm formation by commensal streptococci but inhibit *S. mutans* in the dual species biofilms

**Figure 6:**
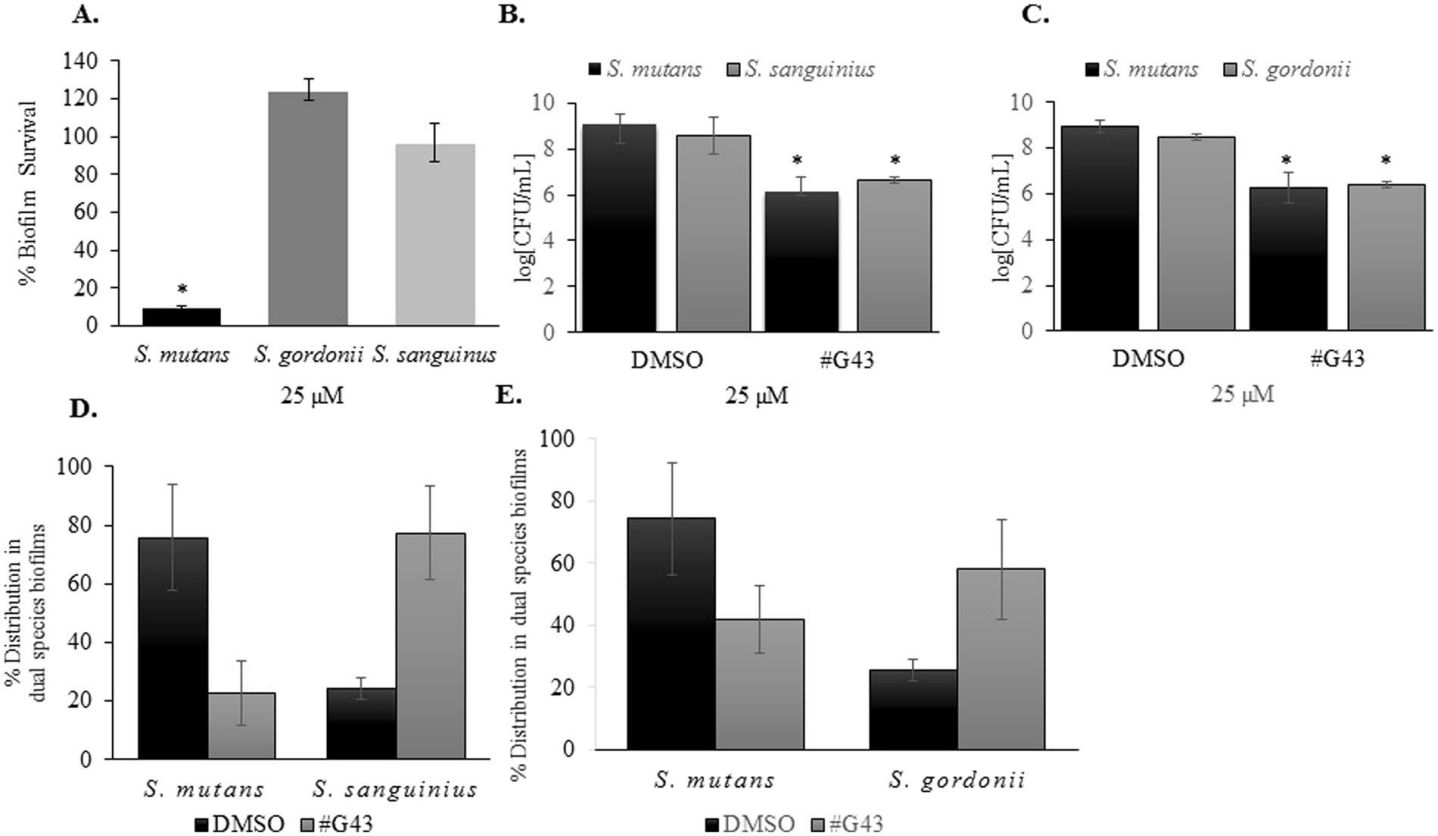
Effects of compound #G43 on commensal single and dual species biofilms. (**A**) *S. mutans*, *S. gordonii*, and *S. sanguinis* were treated with DMSO or 25 µM of compound #G43, and the biomasses of each treated biofilm were quantitated by crystal violet staining and measured at OD_562_. (**B**) The cell viability of dual species biofilms was determined by the numbers of CFU in a logarithmic scale using *S. mutans*, and *S. sanguinis*. (**C**) The cell viability of dual species biofilms was determined by the numbers of CFU in a logarithmic scale using *S. mutans*, and *S. gordonii*. (**D**) Species distribution in dual species biofilms with *S. mutans* and *S. sanguinius*. Bars represent the mean and standard deviations of three independent experiments. (**E**) Species distribution in dual species biofilms with *S. mutans* and *S. gordonii*. Bars represent the mean and standard deviations of three independent experiments. *P < 0.05.

To determine the selectivity of the lead compound toward *S. mutans* biofilm formation over the biofilms of other species, we evaluated effects of the compound on the biofilm formation by two oral commensal bacteria: *S. sanguinis* and *S. gordonii*. No significant difference in *S. sanguinis* biofilm formation was observed between the control group and #G43 treated groups up to 200 µM (Fig. 6A). A slight increase in the biofilm formation by *S. gordonii* was observed when treated with the lead compound. Further, experiments using a dual species model was conducted using *S. mutans* with either *S. sanguinis* (Fig. 6B), or *S. gordonii* (Fig. 6C). We observed a reduction in the overall biofilm formation with the treatment of the compound. Moreover, the lead compound shifted the bacterial composition ratio of commensal streptococcus to *S. mutans* from untreated 1:4 to either 4:1 (Fig. 6D) for *S. sanguinis*, or 3:2 for *S. gordonii* (Fig. 6E). The increase in commensal bacteria by the treatment again suggests that the lead selectively affects *S. mutans* biofilm.

### Docking analysis, the facile synthesis of #G43 and its inactive analog to establish that the ortho primary benzamide moiety is crucial for its potency

To explore the underlying mechanism of #G43’s bioactivity, the compound was docked into the active site of GtfC to elucidate plausible interactions. The top docking pose of #G43 within the GtfC active site revealed several key interactions. The nitro group on the benzothiophene ring interacts with Arg540, the amide linker is within close proximity of Gln592, and pi-pi stacking interactions are observed between Trp517 and the benzene ring. Of particular importance is the interaction of the primary ortho amide group on the benzene ring with Glu515, Asp477, and Asp588. While the mechanism of the glucan formation is not fully understood, Glu515, Asp477, and Asp588 are assumed to function as a nucleophile, a general acid/base catalyst, and a stabilizer of the glucosyl intermediate, respectively^15^. Thus, we hypothesized that this functional ortho amide group is crucial for the binding of the compound to the protein.

**Figure 7:**
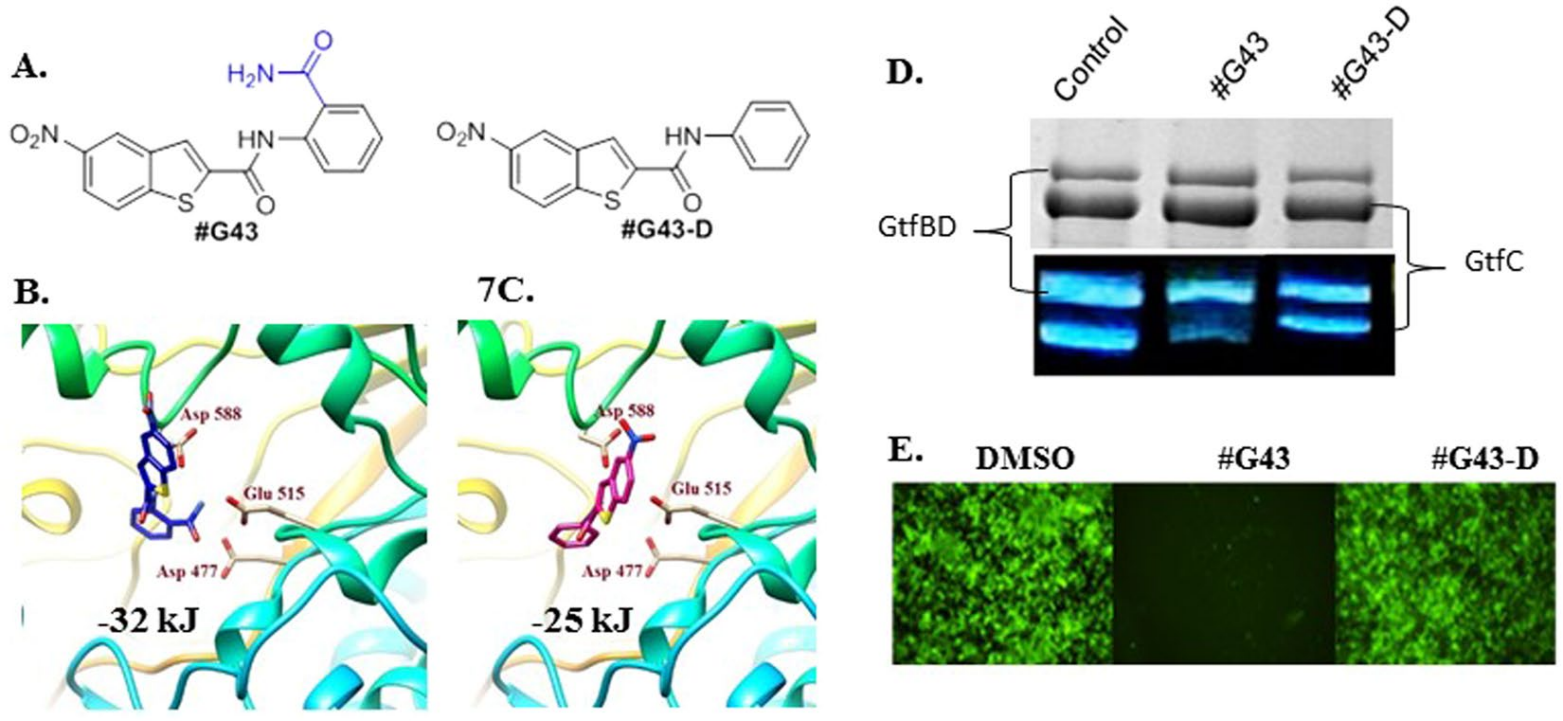
Effects of the lead compound #G43 and its inactive analog #G43-D. (**A**) Chemical structures of lead and its inactive analog. Docking poses of (**B**) Compound #G43 in blue skeleton and (**C**) compound #G43-D in pink skeleton. Three key residue interactions are depicted by displaying residue chains. (**D**) Effects of active and inactive compound on the activity of Gtfs by zymographic assays. Glucan zymographic assays (bottom panel) were performed using SDS-PAGE analysis of Gtfs from culture supernatants of *S. mutans* UA159 incubated with vehicle control DMSO, the synthesized active #G43, and its derivative at 50 µM. SDS-PAGE analysis of Gtfs (top panel) was used as a loading control. (**E**) Fluorescent microscopy images of *S. mutans* UA159 biofilms treated with DMSO control, the synthesized #G43, and its derivative #G43-D at 100 µM. Viable bacterial cells were stained with 2.5 µM Syto9 (green).

In order to test this, we designed an analog (#G43-D) with a 3D structure (Fig. 7A) that does not contain the primary amide group and subjected it to docking analysis, as a theoretical design. This scaffold failed to produce a good docking score in FlexX (greater than −25 kJ/mol) and yielded a weak binding pose (Fig. 7B). Due to the absence of the primary amide group, the scaffold takes on a different orientation and possesses poor interactions with the active site.

The lead compound was re-synthesized in one step using commercially available reagents, anthranlinamide and 5-nitro-1-benzothiophene -2-carboxylic acid, in an excellent yield and fully characterized (see supplemental data). We also synthesized the “inactive” analog (#G43-D) in one step by replacing the anthranilinamide with aniline in the EDAC coupling synthesis. Zymographic analysis consistently showed that the lead compound #G43 drastically reduced the glucan production, especially of those produced by GtfC. However the designed “inactive” compound #G43-D significantly reduced the ability to inhibit the glucan production (Fig. 7D). Additionally, i*n vitro* biofilm assay and fluorescence microscopy revealed that the inactive analog #G43-D did not inhibit *S. mutans* biofilms at concentrations up to 200 µM (Fig. 7E). Binding studies of this analog against GtfB yielded a K_D_ value of 68 µM, compared to a K_D_ value of 3.7 µM by the active analog (see supplemental data). Our data demonstrates that not only is the inhibition of biofilms by selectively targeting Gtfs plausible, but the inclusion of the primary ortho amide group is crucial to maintain potent anti-biofilm activity. Further structure and activity relationship studies are ongoing to improve the potency of #G43.

### #G43 reduced *S. mutans* virulence *in vivo*

To evaluate *in vivo* efficacy of the lead compound #G43, we tested the compound using a rat model of dental caries^27^ (Table 1). All rats from the two experimental groups were colonized with *S. mutans*. The bacterial colonization appears to be reduced in #G43 treated rats, however the reduction did not reach the statistically significant difference when compared with the control group. The buccal, sulcal, and proximal surface caries scores of the treated animals were significantly reduced. These data suggest that the lead small molecule selectively targets virulence factors, Gtfs and Gtf- mediated biofilm formation, rather than a simple inhibition of bacterial growth. Furthermore, the #G43 treated rats did not lose weight over the course of the study in comparison with the control group, suggesting that the compound is not toxic.

**Table 1:** Effects of the lead compound on bacterial colonization and mean caries scores *in vivo*.

Groups	Weight (g)	CFU/ml (10^6^)		Buccal				Sulcal				Proximal			
		MS	BAP	E	Ds	Dm	Dx	E	Ds	Dm	Dx	E	Ds	Dm	Dx
Control	143±13	7.1 ± 0.8	7.2 ± 0.8	16.2 ± 0.2	14.0 ± 0.8	10 ± 0.8	6.6 ± 0.4	24.2 ± 0.6	20.8. ± 0.7	11.8 ± 1	6.2 ± 0.7	8.0 ± 0	4.8 ± 0.5	0.2 ± 0.2	0 ± 0
#G43	144 ± 10	4.4 ± 0.5	4.6 ± 0.6	5.6 ± 0.2	2.8 ± 0.4	1.4 ± 0.5	0.4 ± 0.2	14 ± 0.6	9.6 ± 0.5	3.2 ± 0.5	1.6 ± 0.4	1.6 ± 0.7	0 ± 0	0 ± 0	0 ± 0

Two groups of 6 rats infected with S. mutans UA159, and treated with or without #G43. **Caries Scores:** Buccal: Enamel (E); p < 0.001: Dentinal slight (Ds); p < 0.001: Dentinal moderate (Dm); p < 0.001: Dentinal extensive (Dx); p < 0.001. Sulcal: E; p < 0.001: Ds; p < 0.001: Dm; p<0.001: Dx; p<0.001. Proximal: E; p < 0.001: Ds; p < 0.001: Dm; NS: Dx; NS. **Bacterial colonization:** CFU/ml, NS. **Weights:** NS. NS, no significant difference.

## Discussion

Dental caries is a multifactorial disease, in which *S. mutans* and other cariogenic species interact with dietary sugars to promote virulence. The current marketed therapies for dental caries and other infectious diseases are non-selective and broad spectrum in nature, which compromises the benefit of commensal bacteria in the oral flora. Thus, we have conducted this study to develop novel small molecule inhibitors selective for key virulence factors of *S. mutans*. As Gtfs are crucial for the biofilm formation and the cariogenicity of *S. mutans*, we conducted an *in silico* screening of 500,000 drug-like small molecule compounds targeting GtfC and identified top scored scaffolds for *in vitro* biofilm assays. Seven potent biofilm inhibitors emerged from this study, the lead compound #G43 was further characterized and shown to have anti-biofilm activity through the binding to GtfBC and the inhibition of the activity of GtfBC. The lead compound drastically reduced bacterial virulence in the rat model of dental caries.

In addition, the protein-small molecule binding kinetic analysis of #G43 and GtfBC revealed the lead compound has strong selectivity, it has low micromolar affinity for GtfB and more potent nanomolar affinity for GtfC. Furthermore, compound #G43 selectively inhibited *S. mutans* biofilms in single-species and dual-species biofilm. As the catalytic domain of GtfB and GtfC shares 96% similarity at amino acid sequence level, the selectivity by the compound is remarkable. Since the crystal structure of GtfC/acarbose complex was used for screening and identification of potent lead compounds, this result further demonstrates the validity of this structure-based drug design approach for precision drug discovery. Numerous studies have claimed the identification of natural and synthetic small molecules that inhibit the biofilm formation of *S. mutans* through affecting the gene expression of a variety of biofilm regulatory genes including *gtfs* ^28–31^. Many compounds may have indirect effects on the expression of *gtf*s as they can target different signaling and metabolic pathways. None has been shown to have a direct effect on the activity of Gtfs.

Further investigation through docking analysis of this lead compound identified critical interactions of the ortho primary amide group of the compound with key active site residues of GtfC. An analog that does not contain this functional group lost the ability to inhibit the activity of Gtfs and *in vitro* biofilm formation, demonstrating that these effects are directly related and that the inclusion of the primary ortho amide group is crucial to maintaining potent anti-biofilm activity.

The lead compound contains a nitro group, and typically, nitro groups are not amenable for development of drugs due to the potential of hazardous production of the nitroanion radical, nitroso intermediate, and N-hydroxy derivative^32^. However, this is a concern only for systemic drugs and not for topical applications we intend to carry out. Nevertheless, efforts are underway to optimize the activity and explore the removal of such predicted groups. Further, we were encouraged to find that #G43 did not affect the survival rates of *S. mutans* and two commensal streptococcal species up to 200 µM, and did not significantly affect other common oral bacteria such as *Actinomeyes naeslundii* and *Aggregatibacter actinomycetemcomitans*. The non-toxic feature of #G43 was also evident in the rodent caries models as no weight loss was observed in rats.

A recent study also reported the development of a Gtf inhibitor through a similar approach. The observed potency of our lead compound #G43 is slightly better than the previously reported scaffold^26^. Further #G43 drastically inhibited cariogenicity *in vivo*, but did not significantly inhibit *S. mutans* colonization. This is interesting finding as the compound effectively inhibited the biofilm formation by *S. mutans in vitro*. It is possible that the sampling method skewed our results toward the total numbers of *S. mutans* recovered from the oral cavity rather than only the biofilm bacteria. In addition, *in vivo* inhibition of *S. mutans* glucan production may not be sufficient to inhibit *in vivo* biofilm formation thus bacterial colonization. This would be a desirable outcome as we can inhibit virulence but minimally affect bacterial colonization and demonstrate a virulence-selective therapeutic approach. Moreover, in contrast to the reported compound, #G43 did not significantly affect the expression of Gtfs. We also demonstrate that the lead compound selectively binds to GtfC and GtfB, suggesting the impact on the activity of GtfBC by the direct interaction rather than through downregulation of gene expression of *gtfBC*.

In conclusion, using structure-based design, we have developed a unique low micromolar biofilm inhibitor that targets *S. mutans* Gtfs through binding to key virulence factors, Gtfs. Our compound is drug-like, non-bactericidal, easy to synthesize, and exhibits very potent efficacy *in vivo*. The report disclosed an excellent candidate that can be developed into therapeutic drugs that prevent and treat dental caries.

## Methods

### Structure-Based 3D Database Search

The crystal structure of the complex of GtfC and acarbose (PDB code: 3AIC)^15^ was used for *in silico* screening. The GtfC active site was prepared by selecting residues and cofactors (water and MES) within 6.5 Å of acarbose and then a pharmacophore that consists of Asp588 (H-acceptor) and Gln960 (H-donor) was generated. The reliability of the FlexX/LeadIT package was assessed by virtually generating a 3D structure of acarbose using VEGA-Z, and then by docking the structure into the prepared GtfC active site. This resulting docking generated a comparable binding mode to the experimental data. A large library of about 500,000 small molecules obtained in 3D mol2 format from the free-access ZINC database was used for the *in silico* screening. Docking runs were performed with a maximum allowed number of 2000 poses for each compound. The produced binding energies were ranked according to the highest scoring conformation. Compounds with binding energies better than −20 kJ/mol were selected for further investigation. The structures of top scoring compounds were examined for their bindings inside the GtfC pocket, drug-like properties based on Lipinski’s rules, and for synthetic feasibility.

### Bacterial strains, culture conditions, and chemicals

Bacterial strains, including *S. mutans* UA159 and various Gtf mutants as described below, *S. sanguinis* SK36, and *S. gordonii* were grown statically at 37 °C with 5% CO_2_ in Todd-Hewitt (TH) broth or on THB agar plate, or in chemically defined biofilm medium supplemented with 1% sucrose^33^. *Aggregatibacter actinomycetemcomitans* VT1169 and *Actinomyces naeslundii* T14VJ1 were grown in Tryptic soy broth with yeast extract (TYE).

Small molecule candidates were purchased from either ChemBridge Corporation or Enamine Ltd in USA. Stock solutions were prepared in dimethyl sulfoxide (DMSO) at 10 mM and arrayed in a 96-well format for biological screening.

### *S. mutans* biofilm formation and inhibition assays

Biofilm assays using 96-well flat-bottom polystyrene microtiter plates were performed to evaluate *S. mutans* biofilm formation at various conditions of small molecule inhibitors as described^34, 35^. Each assay was replicated three times. Minimum biofilm inhibitory concentration (MBIC) of compounds was determined by serial dilutions. The most active compounds identified from the tested candidates were selected for further examination.

### Construction of *S. mutans* Gtfs mutants

GtfB, GtfC single mutant, and GtfBC double mutant in which *gtf* was replaced with a kanamycin resistance cassette, *aphA3* (encoding an aminoglycoside phosphotransferase), are gifts from Dr. Robert Burne’s Laboratory, University of Florida, Gainesville, FL. The GtfD mutant was constructed by an overlapping PCR ligation strategy using an erythromycin resistance cassette isolated from the IFDC2 cassette^36^. In brief, a 1-kb DNA fragment upstream of *gtfD* was PCR amplified with a primer pair of GtfD-UpF1 and GtfD-UpR-ldh, while a 1-kb DNA fragment downstream of *gtfD* was PCR amplified with a primer pair of GtfD-DnF-erm and GtfD-DnR1. The erythromycin cassette was PCR amplified with a primer pair of ldhF and ermR. With a primer pair of GtfD-UpF and GtfD-DnR, the overlapping PCR was used to amplify the three fragments that contain overlapping regions (listed in supplemental data). The resulting 2.8-kb *ΔgtfD*/*erm* amplicon was transformed into *S. mutans* UA159, and transformants were selected on THB plates containing erythromycin after 48 h incubation. The GtfBD and GtfCD double mutants were constructed by transformation of the GtfB and GtfC single mutant with the Δ*gtfD*/*erm* amplicon and followed by the selection of kanamycin- and erythromycin-resistant colonies. The in-frame insertion of *erm* in the place of *gtfD* for each mutant allele was verified by DNA sequencing analyses. The mutants were further validated by the production of respective Gtf.

### Inhibition of the activity of Gtfs determined by zymographic assays

A well established zymographic assay was used to determine enzymatic activity of Gtfs^37^. In brief, overnight *S. mutans* UA159 cultures were diluted 1:100 in fresh 5 mL THB. Bacteria were grown to OD_470_ of 1.0, and spun down by centrifugation at 4 °C and culture supernatants were collected and filtered through a 0.22-μm-pore-size filter membrane to remove residual bacterial cells and dialyzed at 4 °C against 0.02 M sodium phosphate buffer (pH 6.8), with 10 μM phenylmethylsulfonyl fluoride (PMSF), followed by a second dialysis against 0.2 mM sodium phosphate containing 10 μM PMSF. After dialysis, 4 mL of samples were concentrated to 40 μL by 100 K Amicon Ultra-4 centrifugal filter (Merk Millipore Ltd.). For electrophoresis and zymographic analysis, 10 μL of each concentrated culture supernatant was applied to 8% SDS-PAGE in duplicate. One gel was used for protein staining with Coomassie blue dye, while the other one was subjected to zymographic assay as described^37^. For zymogram analysis, following electrophoretic separation, gels were washed twice for 15 min each with renaturing buffer containing 2.5% Triton X-100. Gels were then incubated for 18 h at 37 °C with 0.2 M sodium phosphate buffer (pH 6.5) containing 0.2% dextran T70, 5% sucrose, and varying concentrations of the small molecule inhibitors. The reactions were stopped by washing gels with distilled water at 4 °C for 10 min, and digital images of the resultant white opaque glucan bands were visualized against a black background and captured using a digital camera. ImageJ software was used to analyze the intensities of each band, and % of the inhibition by the lead compounds was calculated by comparing the band intensity between lead compounds, and DMSO treated groups.

### Expression and purification of GtfB and GtfC catalytic domains

The DNA fragment coding for either GtfB catalytic (residues 268 aa–1074 aa) or GtfC-catalytic (residues 295 aa–1103 aa) was PCR amplified with primer sets of GtfB-BamH1-F and GtfB-Xho1-R, GtfC-BamH1-F and GtfC-Xhol1-R respectively (Supplemental Table 1) using *S. mutans* genomic DNA as a template. Each amplified fragment was cloned into pET-sumo vector respectively and transformed in *Escherichia coli* BL21(DE3). The recombinant strain grown to OD_600_ = 0.8 in LB medium was induced with 0.1 mM IPTG at 18 °C overnight. Cell lysates prepared form the overnight grown *E. coli* cells were subjected to protein purification using HiTrap^TM^ Column (Ni^2+^ affinity) followed by gel filtration experiments as described^38, 39^.

### Octet Red analysis

Octet full kinetic binding analysis was performed for binding of #G43 to GtfB and GtfC respectively. The rate constant, K_D_, was determined using the Octet® Red96 system (ForteBio, Menlo Park, CA). Phosphate buffer with 3.5% (w/v) DMSO was used. The proteins were captured on dip-and-read Anti-Penta-HIS (HIS1K) Biosensor. These consist of high affinity, high specificity Penta-His antibody from Qiagen pre-immobilized on a fiber optic biosensor. The binding of #G43 at 3-fold serial dilutions in phosphate buffer from 200, 66.6, 22.2, 7.4, 2.5 to 0 µM was assessed. The ForteBio Octet analysis software (ForteBio, Menlo Park, CA) was used to generate the sensorgram and monitor the accuracy of the analysis.

### Cell viability of *S. mutans, S. gordonii, S. sanguinis, Aggregatibacteractinomycetemcomitans* and *Actinomyces naeslundii*

Effects of lead small molecules on cell viability were examined as described^34^. The number of colony-forming units (CFU) per milliliter of each sample treated with selected compounds at different concentrations was enumerated after incubation for 24 h at 37 °C and compared to the values obtained from the DMSO control group. Overnight broth cultures were transferred by 1:50 dilutions into fresh THB medium and were allowed to grow until mid-exponential phase (OD_470_ nm 0.6) before transfer to 96-well plates containing desired concentration of the testing compounds. After 16 h incubation, bacterial growth was measured at OD_470_, and normalized to the DMSO control (100%).

### Growth of commensal and dual-species biofilms

Overnight broth cultures were transferred by 1:50 dilutions into fresh THB medium and were allowed to grow until mid-exponential phase (OD_470_ nm = 0.6) before transfer to 96-well plates. For mono-species biofilms, 1:100 dilution of the individual cultures was added to the 96-well plate containing the desired concentrations of compounds or DMSO. After incubation for 16 h, the biofilms were gently washed with PBS in triplicate and the biofilms were quantified with crystal violet staining. For dual-species biofilms, 1:100 dilution of *S. mutans* was used and 1:10 dilution of the commensal species (*S. sanguinius* or *S. gordonii*) was used as inoculum to seed the 96-well plate containing the desired concentrations of compounds or DMSO. After incubation for 16 h, the biofilms were scratched off with a sterile spatula and suspended in 100 µL of PBS, the biofilm samples were vortexed. To determine the total number of viable bacterial cells (CFU), 100 μl from 16h-dispersed biofilms were serially diluted in potassium phosphate buffer and plated in duplicate on blood agar plates. The commensal species could be differentiated from *S. mutans* due to their characteristic green rings formed around the colonies.

### Rat model of dental caries

*S. mutans in vivo* colonization and virulence were evaluated using a rat model of dental caries as previously described^40–42^. Fischer 344 rats were bred and maintained in trexler isolators. Rat pups were removed from isolators at 20 days of age and randomly assigned into two groups of 6 animals with or without treatment of the potent inhibitor #G43. Rats were then infected with *S. mutans* UA159 for three consecutive days and provided a caries-promoting Teklad Diet 305 containing 5% sucrose (Harlan Laboratories, Inc., Indianapolis, IN) and sterile drinking water ad libitum. One group of rats was then treated with vehicle control while another group was topically treated with the lead compound at 100 µM twice daily for 4 weeks beginning 10 days post infection. Following each treatment, drinking water was withheld for 60 min. Animals were weighed at weaning and at the termination of the experiment. The animals were euthanized, their mandibles excised for microbiological analysis of plaque samples on MS agar plates and blood agar plates and for scoring of caries by the method of Keyes^43^. All experimental protocols were approved by University of Alabama at Birmingham Institutional Animal Care and Use Committee. The methods were carried out in accordance with the relevant guidelines and regulations.

### Synthesis of small molecules

Protocols used to synthesize the lead compound and its subsequent derivatives are described in the supplementary information.

### Statistical Analysis

The analysis of the *in vitro* experimental data was performed by ANOVA and Student’s *t* test using SPSS 11.0 software (SPSS Inc., Chicago, IL).

Statistical significance in mean caries scores, colony-forming units (CFU) per mandible and body weights between two groups of rats was determined by one-way ANOVA with the Tukey–Kramer multiple comparison test using the InStat program (Graphpad Software). Differences were considered to be significant when a value of *P* ≤ 0.05 was obtained.

## Electronic supplementary material

Supplemental
