# Supplementary material for: Structure-Based Discovery of Small Molecule Inhibitors of Cariogenic Virulence
Source: Sci Rep. 2017 Jul 20;7:5974. doi: 10.1038/s41598-017-06168-1 (PMC5519559; doi:10.1038/s41598-017-06168-1)
Supplement: Supplementary file 1 — Supplemental [file 41598_2017_6168_MOESM1_ESM.pdf]

Structure-Based Discovery of Small Molecule Inhibitors of Cariogenic Virulence

Qiong Zhang[1,2§], Bhavitavya Nijampatnam[3§], Hua, Zhang[1], Thao Nguyen[3], Jing Zou[1], Xia Cai[4], Suzanne Michalek[4], Sadanandan E. Velu[3*], and Hui Wu[2*]

1 Department of Pediatric Dentistry, University of Alabama at Birmingham, School of Dentistry, Birmingham, Alabama 35294, USA

2 State Key Laboratory of Oral Diseases, West China Hospital of Stomatology, Sichuan University, Chengdu, Sichuan 610041, P. R. China

3 Department of Chemistry, University of Alabama at Birmingham, 901, 14th Street S. Birmingham, AL 35294, USA

* Corresponding authors: Mailing address, Department of Pediatric Dentistry, University of Alabama at Birmingham, School of Dentistry, Birmingham, Alabama 35294. Phone: (205) 996-2392. Fax: (205)975-4430. E-mail: hwu@uab.edu.
Sadanandan E. Velu, Mailing address, Department of Chemistry, University of Alabama at Birmingham, 901, 14th Street S. Birmingham, AL 35294, USA, Phone: (205) 975-2475, Fax: (205) 934-2543, Email: svelu@uab.edu.

Supplemental data

General Considerations:

Reactions were monitored with thin layer chromatography (TLC), which was done on silica gel plates with fluorescent indicator (Whatmann, silica gel, UV254, 25 μm plates). The TLC spots were observed under UV light with the wavelengths 254 nm and 365 nm. Melting points were determined on a Mel-Temp II melting point apparatus and were uncorrected. Proton nuclear magnetic resonance (^{1H}NMR) and carbon nuclear magnetic resonance (^{13}CNMR) spectra were recorded on Bruker DPX 300 spectrometers using TMS or appropriate solvent signals as internal standard. The values of chemical shift are given in parts per million (ppm) relative to tetramethylsilane and coupling constants (*J*) in Hz. Mass spectra were recorded on an Applied Biosystems 4000 Q Trap instrument. Anhydrous solvents used for reactions were purchased in Sure-Seal™ bottles from Aldrich Chemical Company. Other reagents were purchased from Aldrich, Lancaster or Acros chemical companies and used as received.

Compound #G43

5-nitro-1-benzothiophene -2-carboxylic acid (535 mg, 2.4 mmol), anthranlinamide (300 mg, 2.2 mmol), EDAC (630 mg, 2.2 mmol), and DMAP (26 mg, 0.22 mmol) were dissolved in dichloromethane (15 mL) and the mixture was let stir overnight. TLC examination using10% MeOH in CH_2Cl_2 showed the completion of the reaction. The reaction mixture was diluted with CH_2Cl_2 (20 mL), washed with water (3 × 20 mL), brine (1 × 20 mL) and dried over sodium sulfate. Removal of the drying agent followed by the evaporation of solvent gave the crude product which was filtered and washed with dichloromethane and hexane to afford the pure product as a bright orange solid (654 mg, 80%), mp. 401-402°C; ^{1}H NMR (300 MHz, DMSO-d_6) δ: 7.23(t, 1H, J=7.6 Hz), 7.59 (t, 1H, J=7.8 Hz), 7.96-7.93 (m, 2H), 8.37-8.254 (m, 3H), 8.51(s, 1H), 8.58 (d, 1H, J= 7.8 Hz), 9.03 (d, 1H, J=2.1 Hz), 13.336 (s, 1H). ^{13}C NMR (75MHz, DMSO-d_6) δ: 119.1, 120.2, 120.4, 121.4, 123.23, 124.34, 125.88, 128.8, 132.71, 138.9, 139.4, 145.5, 146.1, 158.9, 171.0.M/Z=325.1 $[M-NH_3]^+$.

Compound #G43-D

5-nitro-1-benzothiophene -2-carboxylic acid (535 mg, 2.4 mmol), aniline (225 mg, 2.2 mmol), EDAC (630 mg, 2.2 mmol), and DMAP (26 mg, 0.22 mmol) were dissolved in CH_2Cl_2 (15mL) and the mixture was let stir overnight. TLC examination using10% MeOH in CH_2Cl_2 showed the completion of the reaction. The reaction mixture was diluted with dichloromethane (20 mL), washed with water (3 × 20 mL), brine (1 × 20 mL) and dried over sodium sulfate. Removal of the drying agent gave the crude product which was filtered and washed with dichloromethane and hexane to afford the pure product as a bright orange solid (350 mg, 52 %); mp. 224-226°C, ^{1}H NMR (300 MHz, $CDCl_3$): δ 13.26 (s, 1H), 8.58 (d, 1H, J=8.4 Hz), 8.49 (s, 1H), 8.10- 7.93 (m, 5H), 7.62-7.48 (m, 3H), 7.19 (t, 1H J=6.9). ^{13}C-NMR (75MHz, $CDCl_3$): δ 171.60, 160.14, 140.99, 140.41, 140.17, 139.43, 133.21, 129.29, 127.24, 126.07, 125.70, 125.61, 123.44, 123.40, 120.49, 119.41. M/Z= $[280.2]^+$.

#G43 ^{1}H NMR (300 MHz, DMSO-d_6)

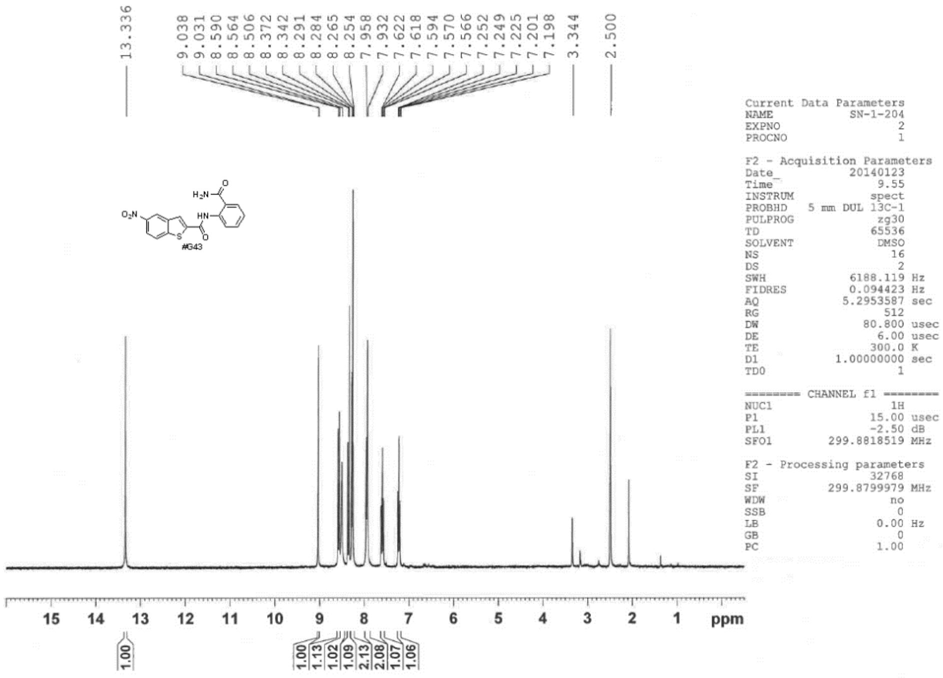

#G43 ^{13}C NMR (75MHz, DMSO-d_6)

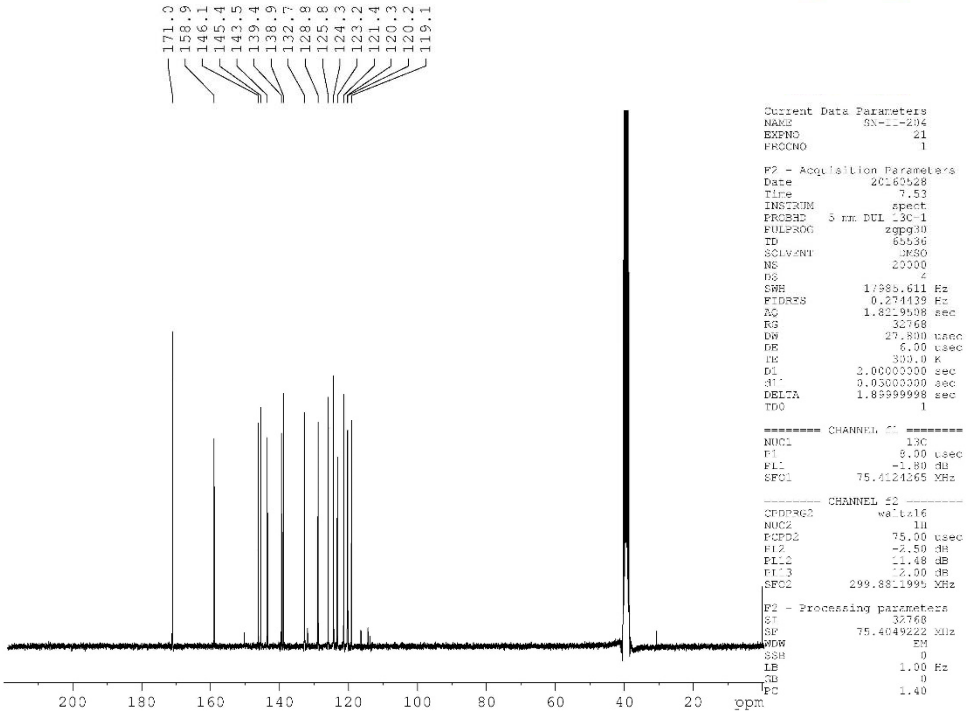

#G43-D ^{1}H NMR (300 MHz, DMSO-d$_6$)

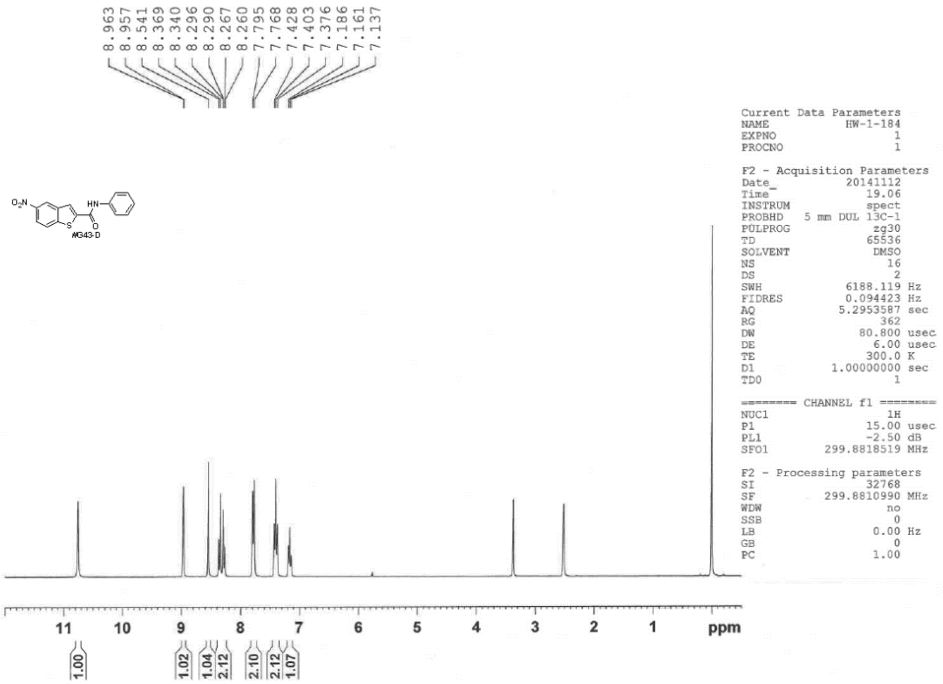

#G43-D ^{13}C NMR (300 MHz, DMSO-d$_6$)

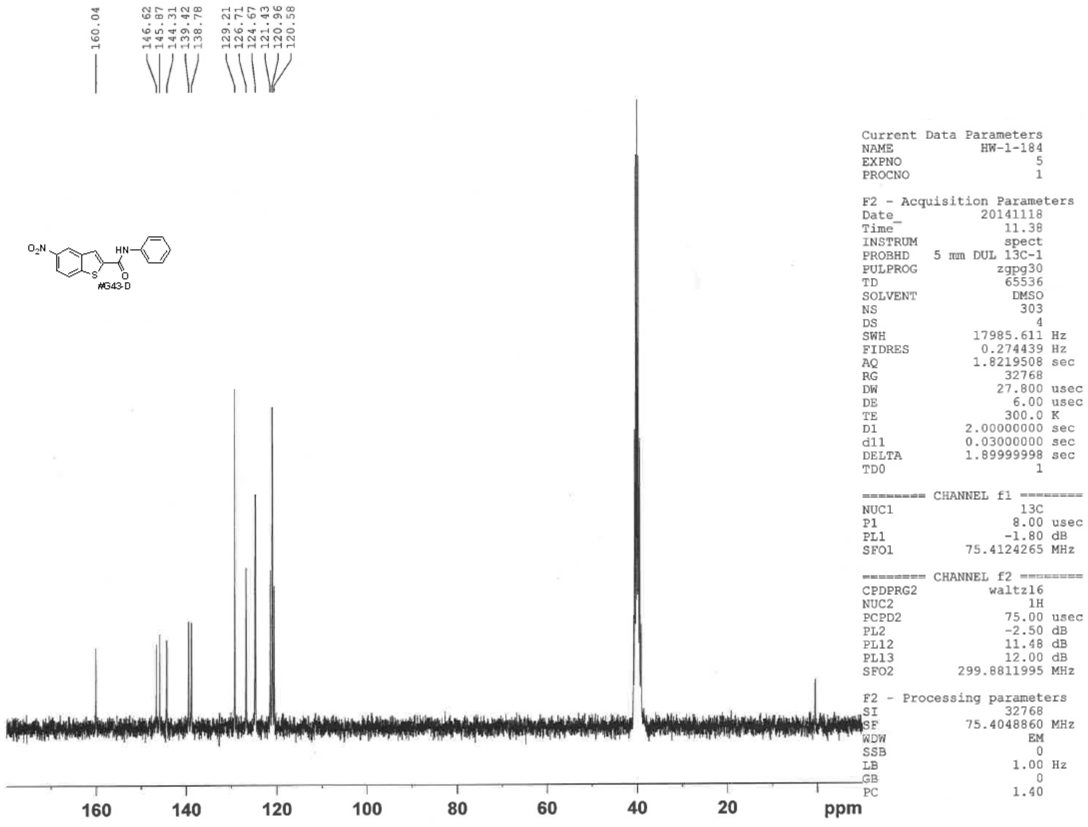

Supplemental Table 1. Primers used in this study

Gene	Primer Sequence (5' - 3') used for overlapping PCR
gtfD-UpF1	TTAGCATGATTGGGGCTGC
gtfD-UpR-ldh	TTGTTCATGTAATCACTCCTTCGATAACATATACGTTACAAAC
gtfD-DnF-erm	GGTATACTACTGACAGCTTCCACTGACATAGCTTAACGTG
gtfD-DnR1	GACAAACATACCTTAGACGC
ldhF	AAGGAGTGATTACATGAACAA
ermR	GAAGCTGTCAGTAGTATACC
Used for real-time RT-PCR	
16s rRNA-UpF	CCTACGGGAGGCAGCAGTAG
16s rRNA-DnR	CAACAGAGCTTTACGATCCGAAA
gtfB-UpF	CATACAGTAACGACAATCAGTAGCTCTA
gtfB-DnR	GTACGAACTTTGCCGTTATTGTCATA
gtfC-UpF	GCCACGGAACAAGCAGTTCTGTAA
gtfC-DnR	TAATACCAATTATTTCCTAAGCTAA
gtfD-UpF2	CACAGGCAAAAGCTGAATTAACA
gtfD-DnR2	GAATGGCCGCTAAGTCAACAG
